# “Himalayan Bridge”: A New Unstable Suspended Bridge to Investigate Rodents' Venturesome Behavior

**DOI:** 10.3389/fnbeh.2021.637074

**Published:** 2021-04-28

**Authors:** Fabiana Festucci, Clelia Buccheri, Anna Parvopassu, Maurizio Oggiano, Marco Bortolato, Giovanni Laviola, Giuseppe Curcio, Walter Adriani

**Affiliations:** ^1^Center for Behavioural Sciences and Mental Health, Istituto Superiore di Sanità, Rome, Italy; ^2^Department of Biotechnological and Applied Clinical Sciences, University of L'Aquila, L'Aquila, Italy; ^3^European Mind and Metabolism Association, Rome, Italy; ^4^Department of Pharmacology and Toxicology, College of Pharmacy, University of Utah, Salt Lake City, UT, United States

**Keywords:** dopamine, risk-taking behavior, wild-type, knock-out, adolescence, bridge length, bridge height

## Abstract

While both risk-taking and avoidant behaviors are necessary for survival, their imbalanced expression can lead to impulse-control and anxiety disorders, respectively. In laboratory rodents, the conflict between risk proneness and anxiety can be studied by using their innate fear of heights. To explore this aspect in detail and investigate venturesome behavior, here we used a “Himalayan Bridge,” a rat-adapted version of the suspended wire bridge protocol originally developed for mice. The apparatus is composed of two elevated scaffolds connected by bridges of different lengths and stability at 1 m above a foam rubber-covered floor. Rats were allowed to cross the bridge to reach food, and crossings, pawslips, turnabouts, and latencies to cross were measured. Given the link between risky behavior and adolescence, we used this apparatus to investigate the different responses elicited by a homecage mate on the adolescent development of risk-taking behavior. Thus, 24 wild-type (WT) subjects were divided into three different housing groups: WT rats grown up with WT adult rats; control WT adolescent rats (grown up with WT adolescents), which showed a proclivity to risk; and WT rats grown up with an adult rat harboring a truncated mutation for their dopamine transporter (DAT). This latter group exhibited risk-averse responses reminiscent of lower venturesomeness. Our results suggest that the Himalayan Bridge may be useful to investigate risk perception and seeking; thus, it should be included in the behavioral phenotyping of rat models of psychiatric disorders and cognitive dysfunctions.

## Introduction

The decision-making process that leads individuals to choose between different beneficial and harmful options is at the heart of everyday life. When faced with these choices, subjects can evaluate the likely outcome of their behavior by weighing risks and rewards, and ultimately decide whether to engage in venturesome or conservation behavior. Abnormal risky decision-making, which is associated with dysregulated dopamine receptor expression, is a characterizing feature of many psychiatric disorders, such as impulse-control disorders, attention deficit hyperactivity disorder (ADHD), schizophrenia, major depression, addiction, and Parkinson's disease (Bechara et al., 2001; Ernst et al., 2003; Ludewig et al., 2003; Taylor Tavares et al., 2007; Kobayakawa et al., 2008).

Crossing a suspended bridge is considered venturesome not only because of the risk of falling but also because of the fear of heights. The acquisition of fear of heights is probably based more on tactile than visual cues because the rat is myopic and with an undifferentiated floor would be unable to make graded depth judgments; it is also likely that depth is judged in an “all-or-none” manner: low enough and safe vs. high enough to evoke fear (File et al., 1998). The fact that the fear of heights is rapidly acquired during initial exposure to the apparatus suggests that there is a genetic, innate predisposition to develop this fear. Conditioned fear, based on associating cues/contexts to unescapable aversive stimuli, or instrumental punishment, based on negative reinforcement to cancel an unwanted action with its punishment, has been used to model phobia; however, simple exposure to frights or innately scary situations has not yet been used to prove useful animal models of phobia. Therefore, this rapidly acquired fear of heights using a naturalistic test situation may prove more useful (Klein, 1980). We presently challenge rats with acute fear induced by the feeling of danger, and therefore this task is not directly comparable to any conditioned-fear task. As far as phobic behavior is concerned, however, Zelli et al. (2020) recently developed a “sudden fright” task that proved useful to highlight a phobic phenotype in dopamine transporter (DAT)-heterozygous rats. This feeling of danger causes excessive and maladaptive avoidance that contributes to the development and maintenance of anxiety disorders and prevents the extinction of fearful responses in humans (Craske et al., 2009; Lovibond et al., 2009) and rodents (Muigg et al., 2008).

The elevated plus maze (EPM) is the gold standard to assess approach–avoidance behavior in rodents, but a homologous test in humans is lacking. For this reason, Biedermann et al. (2017) translated the EPM test into a human paradigm, using a novel task in mixed reality through a combination of virtual and real-world elements. Such task allows tracking of approach–avoidance behavior that is ecologically and ethologically valid. Firstly, experimenters observed a high immersion in the mixed-reality test: participants often gasped at the beginning of the procedure and moved precariously and slow on open arms. Secondly, on a physiological level, the EPM stimulated the sympathetic nervous system; this has been demonstrated by a rise in skin conductance level (SCL), heart rate, and respiration rate. On a behavioral level, participants spent most of the time in the safe compartments of the EPM; on a subjective level, after the experiment, participants stated that they had felt more anxious on open vs. closed arms and center (safe zones). Thirdly, the authors found a high correlation between subjective and behavioral outcomes. Lastly, the authors found significant associations of behavioral measures with trait measures of acrophobia and sensation-seeking (Biedermann et al., 2017).

The goal of our study was to develop a new structure to be able to study the proclivity of rats in risk-taking. For our purpose, we were inspired by the work by Bortolato et al. (2009). To investigate the impact of monoamine oxidase (MAO) B deficiency on the emotional responses elicited by environmental cues, these authors tested MAO B knockout (KO) mice in a set of behavioral assays capturing different aspects of anxiety-related manifestation, including the wire-beam bridge test. Low levels of platelet MAO activity have been strongly associated with features of the behavioral disinhibition spectrum including impulsivity, sensation-seeking, and risk-taking. To capture these elements, the authors measured the animals' proclivity to cross an unrailed flexible bridge suspended over a 30-cm-deep gap to reach a food reward. MAO B KO mice exhibited a significantly shorter latency to access the bridge. In the time before accessing the bridge, MAO B KO mice engaged in a significantly higher sniffing frequency compared to wild-type (WT) mice. These results provide further support that MAO B KO mice display greater impulsivity, sensation-seeking, and risk-taking behaviors than WT mice (Bortolato et al., 2009). The same apparatus was used by the same group to study the combined effect of reserpine (RES), a monoamine-depleting agent, and pramipexole (PPX), a D_2_ and D_3_ dopamine receptor agonist, on rats' impulsive behavior. The rationale of this study relies on the hypothesis that PPX would stimulate sensation-seeking in a context of dopamine depletion. The authors found that the association of RES and PPX does not augment the proclivity of rats to cross the bridge to obtain a reward. This result suggests that the effect of RES and PPX does not reflect a generalized increase in impulsivity and venturesomeness (Orrù et al., 2020). The advantage of this task is that the animals may feel the risk of falling solely because the bridge bends. On the EPM, the platform is stable, and the subjects instinctively know that their risk of falling is minimal as long as they stay on the platform. In contrast, the present paradigm imposes a perception of current (rather than potential) danger, which requires the enactment of coping strategies.

On a neurobiological level, several studies tried to identify the brain regions responsible for the decision-making behavior. Salamone et al. (1994) found that dopamine depletion in the nucleus accumbens biased rats toward making less effortful choices in a T-maze cost–benefit procedure. Walton et al. (2002) later showed that relatively large lesions of the medial pre-frontal cortex in rats also reduced the likelihood of effortful choices. This same group also demonstrated that relatively small lesions of the anterior cingulate cortex decreased effortful choices, whereas lesions to the prelimbic/infralimbic cortex and orbitofrontal cortex did not (Walton et al., 2005). Finally, Floresco and Ghods-Sharifi (2007) showed that the amygdala may also serve as a locus of effort-based decision-making in the brain, since bilateral inactivation of the basolateral amygdala concurrent with inactivation of the contralateral anterior cingulate cortex decreases effortful behavior driven by a food reward. All brain regions currently implicated in effort-based decision-making utilize dopamine released from neurons in the ventral tegmental area as a neurotransmitter: this observation suggests a central role for dopamine in effort-based decision-making. Despite this, the specific dopamine receptor subtypes required for such responses have not been identified (Bardgett et al., 2009).

The DAT is involved in the uptake of dopamine released into the extracellular space; deficiency of DAT function can lead to a hyperdopaminergic phenotype, altering gratification, cognitive, emotional, and motor functions (Salatino-Oliveira et al., 2018). In this context, a new rat model has been developed. In these animals, the gene encoding DAT has been disrupted by using zinc finger nuclease technology: bearing a truncated DAT (DAT-trunk) protein, KO (DAT-KO) rats develop normally but weigh less than heterozygous (HET) and WT rats. DAT-KO rats display elevated locomotor activity and restless environmental exploration associated with a transient anxiety profile, as well as a pronounced stereotypy and compulsive-like behavior (Adinolfi et al., 2019).

In this experiment, a suspended bridge (named “Himalayan” to underscore its similarity to the rope bridges extending over canyons and valleys across Nepal) was exploited to assess the potential difference in novelty-seeking and venturesomeness using a rat model for deviant adolescent trajectories. This was achieved by housing normal WT adolescent rats with either WT adult rats or with DAT-trunk adult rats. These housing arrangements were intended to represent a continuum of adolescent rearing and development ranging from “normal” (adolescent rats reared with adolescent peer rats) and “slightly abnormal” (adolescent rats housed with adult WT rats) to “highly abnormal” (adolescent rats reared with behaviorally atypical adult-DAT-trunk rats) (see Parvopassu et al., 2021). The goal of this study was to investigate how the developing behavior of adolescent WT rats was influenced by the DAT-trunk adult's actions after a period. During adolescence, rats develop behavioral skills through social interaction and play with conspecifics. Given the restricted behavioral profile expressed by DAT-trunk rats, consisting of hyperactivity and stereotypy (Cinque et al., 2018), we hypothesized that growing WT rats would have no way to develop behavioral skills due to a narrowed and altered interaction. However, since DAT-trunk cagemates were also adult, there was the need for a third “intermediate” group, which was housed with an adult but of a WT genotype. Adult WT rats express a normal behavioral repertoire but are however less prone to play with adolescents, whose development may thus take a somewhat altered trajectory. In both cases, such poor social interaction might interfere with the proneness to express, later, appropriate coping skills during a challenge. Influence on them was recently shown to yield a depressive and compulsive phenotype (Parvopassu et al., 2021).

In this way, we were able to assess whether companion affects the risk-taking proclivity, regardless of the genotype. Studies conducted in humans and other mammalian species have reported that adolescents often exhibit more risk-taking behavior than adults (Doremus-Fitzwater et al., 2010; Sturman and Moghaddam, 2011). Such differences are likely driven by neurobiological and hormonal changes that affect cognition and motivation (Doremus-Fitzwater et al., 2009). It has been suggested that these typical adolescent alterations are evolutionarily adaptive in that they cause animals to leave the nest, to mate, acquire resources (Steinberg and Belsky, 1996; Spear, 2010), and, ultimately, facilitate the transition from juvenile period to adulthood (Gore-Langton et al., 2020). It has been postulated that an imbalance between the early-maturing reward and later-maturing cognitive control systems may lead to the elevated impulsive and risk-taking behaviors of adolescents (Ernst et al., 2006; Doremus-Fitzwater et al., 2010; Sturman and Moghaddam, 2011). For these reasons, it is our opinion that WT adolescent rats grown up with WT adolescent rats will be more likely to take the risk of falling to get the food.

The apparatus consisted of an arrival point and a departure point linked by metal bridges of different lengths. This structure was placed 1 m above the floor, which was covered with foam rubber to avoid damage to subjects in case of a fall. Subjects had to cross the bridge to reach the arrival point where a food reward was available.

## Materials and Methods

### Subjects

The generation of Wistar-Han DAT-KO rats was previously described elsewhere (Leo et al., 2018). The colony was maintained in a heterozygous-heterozygous breeding fashion; these animals were intercrossed for >10 generations at *Istituto Italiano di Tecnologia* (ITT, Genoa, Italy). Some progenitors were shipped to *Istituto Superiore di Sanità* (ISS, Rome, Italy), where male DAT-KO rats (and their DAT-WT siblings) were bred with outbred Wistar-Han WT females (Charles River, Italy). As such, we obtained a G0 of founders (namely, heterozygous and WT G0 subjects, respectively). From that step onward, two parallel lines were maintained with a heterozygous-heterozygous vs. a WT-WT breeding fashion. Present subjects are G4 of our ISS colony. All rats were born by “typical” breeding. In particular, WT rats were offspring by WT mothers bred with WT fathers, while HET rats were offspring by HET mothers bred with HET fathers. Animals were maintained under a 12-h reverse dark–light cycle (lights off at 7:00 a.m.) in a temperature- and humidity-controlled environment (T 21 ± 1°C, relative humidity 60 ± 10%) with food (ALTROMIN-R, Rieper SpA, Vandoies, Italy) and water provided *ad libitum*. All test procedures were performed during the dark phase of the cycle.

The experimental group consisted of 24 subjects (eight subjects per group): for the cagemates, 16 rats were male adults, eight rats were male adolescents (respectively born in February 2019 and in March 2019, all weaned at postnatal day 24), and they all weighed around 300–400 g at the beginning of the habituation session. Subjects were at least 3–4 months old at the beginning and no more than 4–5 months old at the end of the procedure.

Subjects were housed in pairs in Makrolon cages. The first group consisted of eight WT rats grown up with truncated-DAT rats (DAT^trunk^ adult companion); the second group consisted of eight WT rats grown up with WT rats (WT adult companion); the third group consisted of eight WT adolescent rats grown up with WT adolescent rats (WT peer companion).

### Apparatus and Procedure

Our experiment aimed to observe the behavior differences of WT rats grown in different conditions and faced with a suspended-bridge task.

The apparatus consisted of two plastic boxes (34 × 24 × 25 cm each) with black floor and sidewalls, one of which was the starting point (A) and the other one, containing pieces of food pellet, was the endpoint (B), connected by a steel bridge that had a 5-cm width.

**Figure 1:**
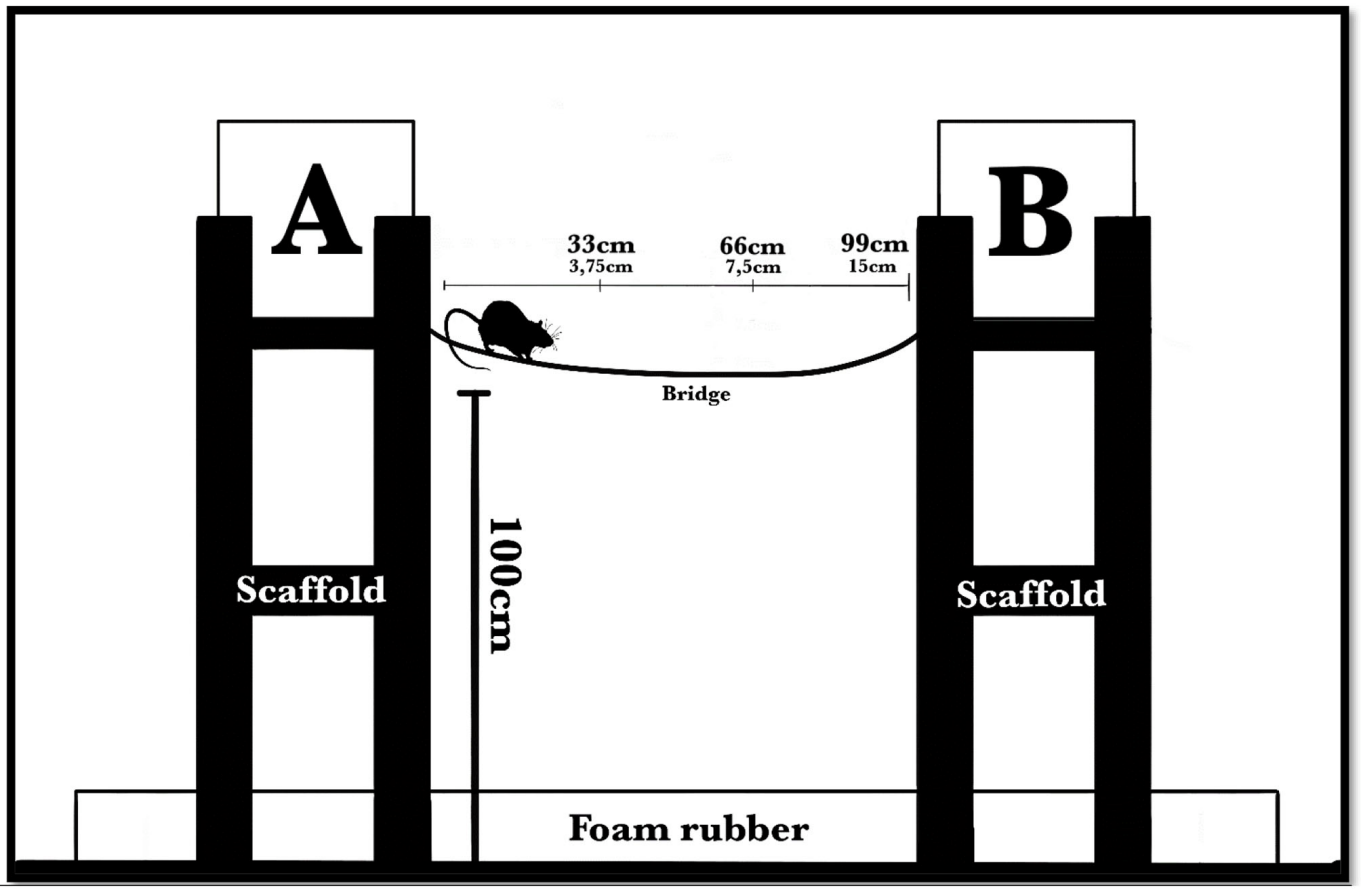
The apparatus consisted of two plastic boxes (34 × 24 × 25 cm each leaning on the long side), one of which was at the starting point **(A)** and the other at the end point (**B**, containing pieces of food pellet), connected by a steel wire-mesh bridge that had a 5-cm width. Each box was placed on a wooden scaffold 1 m above the ground, and the room floor was covered with foam rubber to avoid damage to subjects in the event of a fall. The distance between the boxes depended on the length of the bridge used in specific phases of the procedure: first, 33 cm long, with a 3.75-cm difference in level (midway bending); second, 66 cm long, with a 7.5-cm difference in level; third, 99 cm long, with a 15-cm difference in level. Lastly, the 99-cm bridge was made unstable by means of short chains suspending it to the end scaffold; eventually, we added a “gap” between the bridge and the end scaffold, requiring a little jump as the last step.

Each box was placed on a wooden scaffold 1 m above the ground, and the room floor was covered with foam rubber to avoid damage to subjects in the event of a fall. None of the subjects ever fell. The distance between the boxes depended on the length of the bridge used in specific phases of the procedure (Figure 1).

To build the bridges, we used a wire-mesh metallic grid, cutting some of the internal links, so the wire-mesh became composed of rectangles (5 × 3.5 cm each). We used three stable bridges with different lengths: first, 33 cm long, with a 3.75-cm difference in level due to bending; second, 66 cm long, with a 7.5-cm difference in level; third, 99 cm long, with a 15-cm difference in level. Lastly, we made the 99-cm bridge unstable by means of short suspending chains, so that it oscillated under the animal's weight; eventually, we added a “gap” between the bridge and the endpoint to investigate the subjects' last “step” behavior in that particular situation. Each subject performed the task at least once on each bridge.

**Figure 2:**
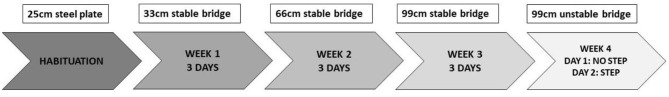
The procedure was conducted 3 days per week for 4 weeks on Tuesday, Wednesday, and Thursday. Animals were food-deprived at homecage on Monday morning, and food was available *ad libitum* on Thursday evening. Initially, subjects underwent 2 days of habituation. They had a 25-cm steel plate to cross from “A” to “B.” During the 1st week, subjects performed on the 33-cm bridge. During the 2nd week, subjects performed on the 66-cm bridge. During the 3rd week, subjects performed on the 99-cm stable bridge. During the 4th week, subjects performed on the 99-cm unstable bridge. In the latter case, during the 2nd day of exposure, we added a space between the bridge and the arrival point to increase the subjects' perception of danger. Moreover, in this way, the bridge swung more during the last “step”.

The procedure was conducted 3 days per week, for 4 weeks, on Tuesday, Wednesday, and Thursday. Each rat performed one trial per day. Animals were food-deprived at homecage on Monday morning and food was available *ad libitum* on Thursday evening. From Monday until Thursday, rats could eat only by reaching end box, to increase motivation for food. Initially, subjects underwent 2 days of habituation: they had a 25-cm steel plate to cross from “A” to “B.” During the 1st week, subjects performed on the 33-cm bridge; during the 2nd week, subjects performed on the 66-cm bridge; during the 3rd week, subjects performed on the 99-cm stable bridge; during the 4th week, subjects performed on the 99-cm unstable bridge. In the latter case, during the 2nd day of exposure, we added a space between the bridge and the arrival point to increase the subjects' perception of danger. Moreover, in this way, the bridge swung more during the last “step.” See Figure 2 for the timeline.

For each trial, subjects were placed in box “A” and remained in the apparatus for a total of 5 min. During the trials, observations considered the complete crossings of the bridge (crossings), the slips during the crossing (pawslips), when the subject returned to the starting point without completing the initiated crossing (turnabout), the time elapsed between the introduction of the subject into the apparatus and the first crossing (latency).

The behaviors were scored by a blind observer. The observer scored every behavior (see Tables 1–3 for the complete data), such as the number of crossings, pawslips, and turnabout, also tracking their times with a stopwatch (i.e., latencies for the crossings).

**Table 1:** Mean (±SEM) number of the performances on the different bridges in Group 1 (WT rats grown up with DAT^trunk^ rats).

**Bridge**	**Crossings**	**Pawslips**	**Turnabouts**	**Latencies (s)**
33 cm, stable	4.12 ± 5.51	0.13 ± 0.35	0.38 ± 0.74	106.20 ± 47.12
66 cm, stable	3.25 ± 3.15	0.00 ± 0.00	0.13 ± 0.35	36.50 ± 29.78
99 cm, stable	2.37 ± 2.20	0.00 ± 0.00	0.00 ± 0.00	58.25 ± 44.16
99 cm, unstable	0.78 ± 0.77	0.13 ± 0.35	1.25 ± 1.28	10.00 ± 8.48
99 cm, unstable w/ gap	0.38 ± 0.74	0.50 ± 0.75	1.25 ± 1.28	11.00 ± 8.48

**Table 2:** Mean (±SEM) number of the performances on the different bridges in Group 2 (WT rats grown up with WT rats).

**Bridge**	**Crossings**	**Pawslips**	**Turnabouts**	**Latencies (s)**
33 cm, stable	4.50 ± 3.07	0.38 ± 0.35	0.38 ± 0.74	97.78 ± 44.89
66 cm, stable	3.50 ± 3.33	0.00 ± 0.00	0.62 ± 0.91	40.08 ± 28.02
99 cm, stable	3.13 ± 2.29	0.13 ± 0.35	0.13 ± 0.35	74.33 ± 36.89
99 cm, unstable	1.25 ± 0.88	0.38 ± 0.51	0.88 ± 0.64	39.00 ± 23.70
99 cm, unstable w/ gap	0.75 ± 0.88	0.38 ± 0.51	1.50 ± 1.92	144.75 ± 80.78

**Table 3:** Mean (±SEM) number of the performances on the different bridges in Group 3 (WT adolescent rats grown up with WT adolescent rats).

**Bridge**	**Crossings**	**Pawslips**	**Turnabouts**	**Latencies (s)**
33 cm, stable	4.50 ± 3.16	0.13 ± 0.35	0.50 ± 0.75	113.42 ± 56.27
66 cm, stable	4.38 ± 3.24	0.00 ± 0.00	0.63 ± 0.51	31.35 ± 18.12
99 cm, stable	4.13 ± 3.60	0.13 ± 0.35	0.13 ± 0.35	63.85 ± 56.30
99 cm, unstable	1.62 ± 1.30	0.00 ± 0.00	0.63 ± 0.74	32.00 ± 55.03
99 cm, unstable w/ gap	1.38 ± 0.91	0.75 ± 0.88	1.38 ± 0.91	53.67 ± 39.64

### Statistical Analysis

We ran three different analyses to investigate three different conditions.

First, we investigated the subjects' performances (crossings, pawslips, turnabouts, and latencies) in the three different “distance” conditions (33-, 66-, 99-cm stable bridges) using a repeated-measure ANOVA with a 3 × 3 × 2 design: “companion” (three levels: DAT^trunk^ companion, WT adult companion, WT peer companion) was a between-subjects factor; all the factors were within-subjects: “bridge” (three levels: 33 vs. 66 vs. 99 cm), “day” (two levels: day 1 vs. day 2).

Then, we investigated the subjects' performances (crossings, pawslips, turnabouts, and latencies) in the two different “stability” conditions (during the 1st day on the 99-cm stable bridge and during the 1st day on the 99-cm unstable bridge) using a repeated-measure ANOVA with a 3 × 2 design: “companion” (three levels: DAT^trunk^ companion, WT adult companion, WT peer companion) was a between-subjects factor; the within-subjects factor was “stability” (two levels: stable vs. unstable).

Eventually, we investigated the subjects' performances (crossings, pawslips, turnabouts, and latencies) in the “step” condition (during the 1st day vs. during the 2nd day on the 99-cm unstable bridge) using a repeated-measure ANOVA with a 3 × 2 design: “companion” (three levels: DAT^trunk^ companion, WT adult companion, WT peer companion) was a between-subjects factor; the within-subjects factor was “step” (two levels: no-step vs. step).

A *p*-value < 0.05 was considered significant. The range between 0.05 < *p* < 0.10 was considered a significant trend. Tukey honestly significant difference (HSD) *post-hoc* test was then performed.

### Ethical Note

All experimental procedures have been approved by the ISS animal welfare survey board on behalf of the Italian Ministry of Health (formal license 937/2018-PR and 1008/2020-PR for project D9997.110, delivered to W. Adriani). Procedures were carried out in close agreement with the directive of the European Community Council (2010/63/EEC) and with Italian law guidelines. All efforts have been made to minimize the suffering of the animals and to use as few animals as possible, according to the 3Rs principle.

## Results

### Stable Bridges Differing for Distance

For “crossings” and “pawslips,” the ANOVA does not show any significant effects. For “turnabout,” a significant trend was presented (*p* < 0.08; *F*_2,42_ = 2.777) for “bridge” due to increasing lengths. Pairwise comparisons show that subjects returned to the starting point significantly more (*p* < 0.05) when they faced the 66-cm bridge than the 99-cm one. The ANOVA shows a significant effect for the “day” (*p* < 0.001; *F*_1,21_ = 21.295). Subjects returned to the starting point significantly more during each 1st day of the task weeks than during the second one. The ANOVA shows a significant interaction for “bridge ^*^ day” (*p* < 0.05; *F*_2,42_ = 3.500). The ANOVA does not show any between-subjects significant effect.

For “latency,” the ANOVA shows a significant effect (*p* < 0.001; *F*_2,30_ = 13.689) for “bridge” due to increasing lengths. Pairwise comparisons show that subjects cross the 66-cm bridge significantly earlier (*p* < 0.001) than the 33-cm one. Moreover, pairwise comparisons show significant trends for the 99-cm bridge. Indeed, subjects cross the 99-cm bridge earlier than the 33-cm one (*p* < 0.09) but *later* than the 66-cm bridge (*p* < 0.09). The ANOVA shows a significant effect for the “day” (*p* < 0.001; *F*_1,15_ = 41,934). During each 2nd day, subjects cross the bridges significantly earlier than during each 1st day. Finally, the ANOVA shows an interaction significant effect for “bridge ^*^ day” (*p* < 0.05; *F*_2,30_ = 4.859). The ANOVA does not show any between-subjects significant effect.

### Longest Bridges Differing for Stability

For “crossings,” the ANOVA shows a significant “stability” effect (*p* < 0.05; *F*_1,21_ = 5.402). Subjects cross significantly more the stable bridge than the unstable one. The ANOVA does not show any between-subjects significant effect.

**Figure 3:**
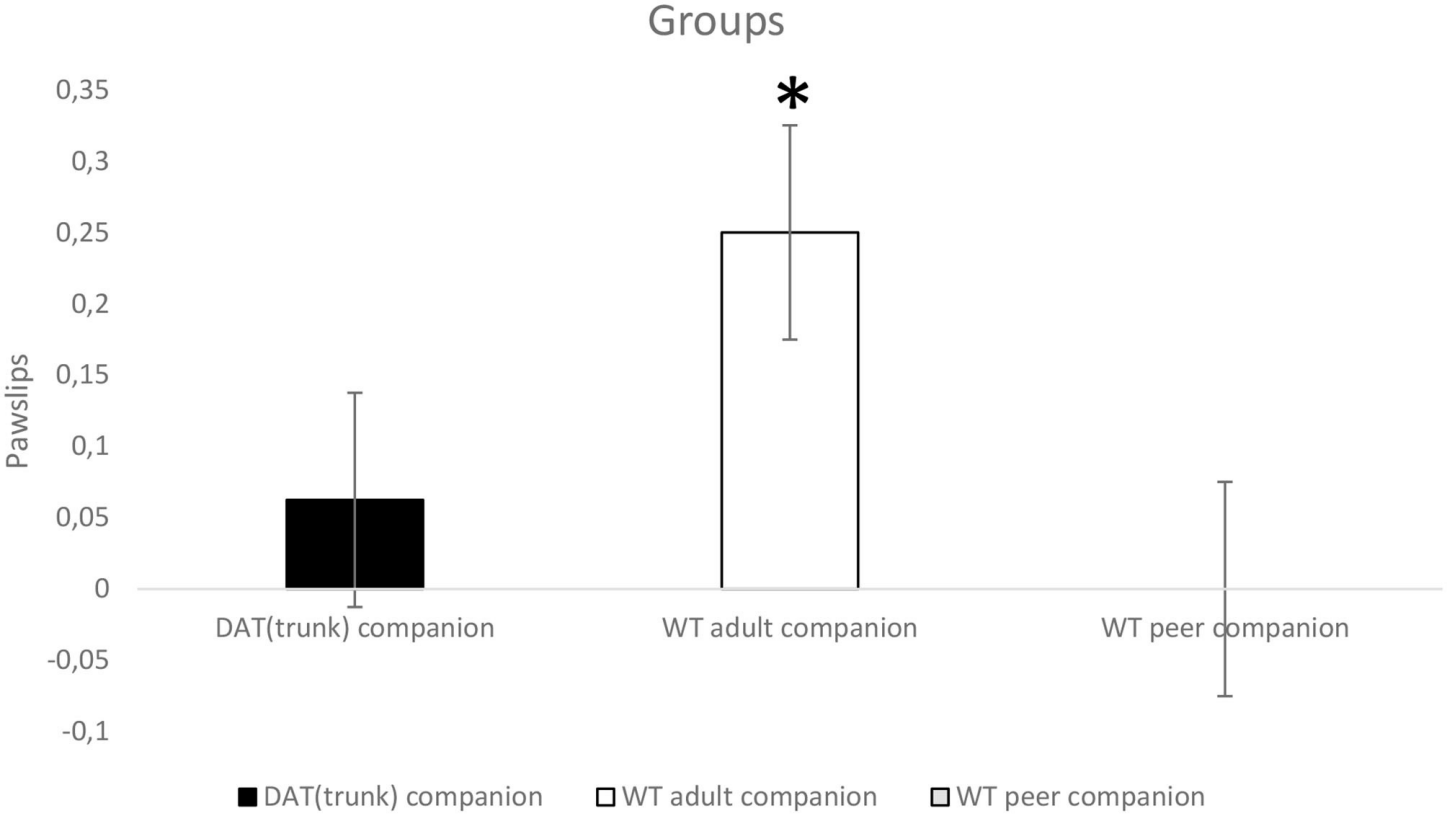
Wild-type (WT) rats grown up with adult WT rats (white) (*n* = 8) slip significantly more (*p < 0.05) than WT adolescent rats grown up with WT adolescent rats (gray) (*n* = 8) when they faced up both the 99-cm stable bridge and the 99-cm unstable bridge.

For “pawslips,” the ANOVA does not show a within-subjects significant effect, but it shows a between-subjects significant effect (*p* < 0.05; *F*_2,21_ = 3.957). Tukey HSD *post-hoc* test shows that WT with adult companion rats slip significantly more (*p* < 0.05) than WT peer companion rats which nearly never slip at all (Figure 3).

For “turnabout,” the ANOVA shows a significant “stability” effect (*p* < 0.001; *F*_2,21_ = 18.485). Subjects returned to the starting point significantly more when they faced the unstable bridge than the stable one. The ANOVA does not show any between-subjects significant effect.

For “latency,” the ANOVA shows a significant “stability” effect (*p* < 0.05; *F*_1,14_ = 6.920). Indeed, subjects cross the unstable bridge significantly *earlier* than the stable one. The ANOVA does not show any between-subjects significant effect.

### Unstable Bridges Differing for the Last Step

**Figure 4:**
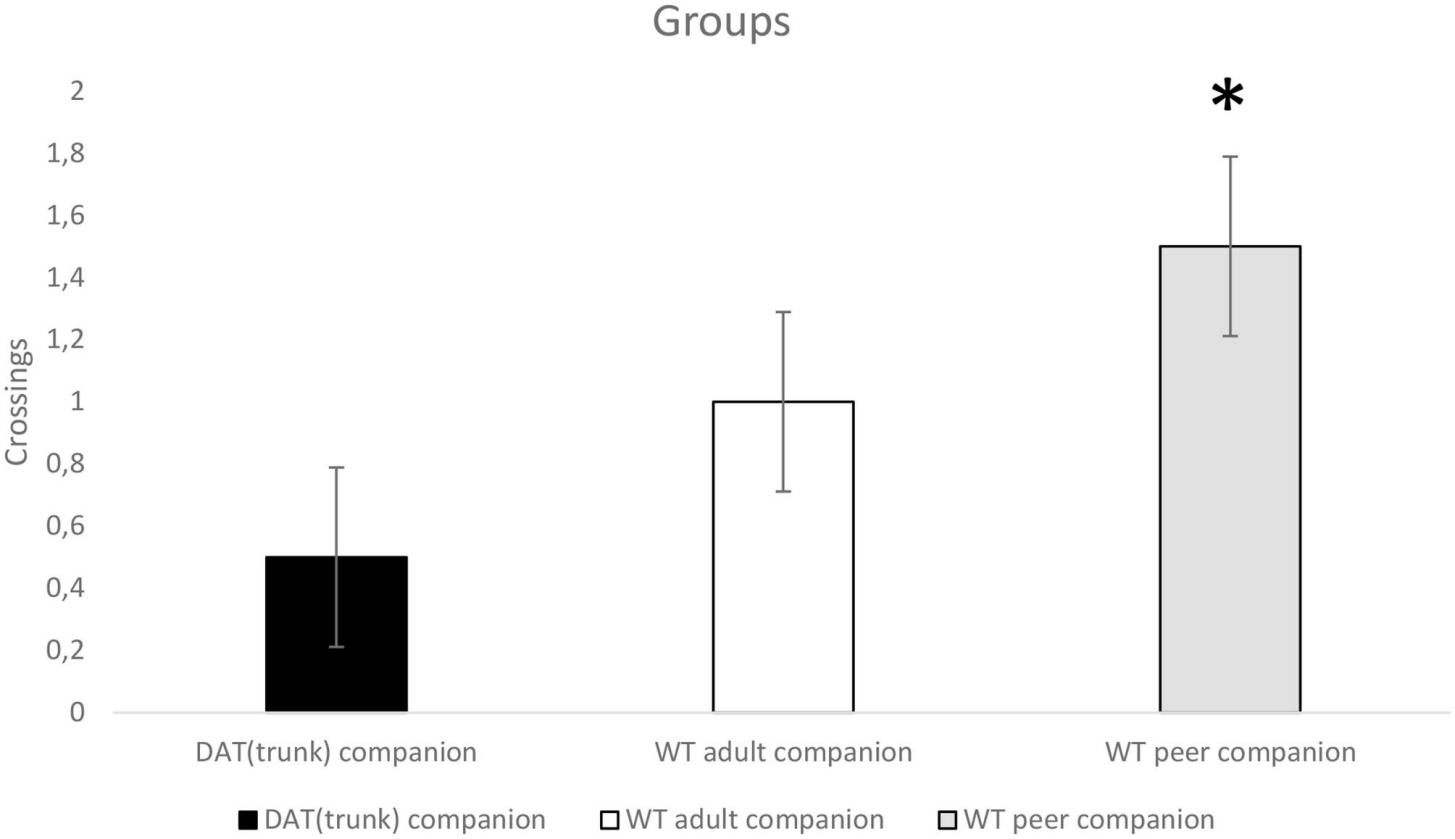
Wild-type (WT) adolescent rats grown up with WT adolescent rats (gray) (*n* = 8) cross more (*p < 0.06) than WT rats grown up with DAT^trunk^ rats (black) (*n* = 8) when they faced up both the 99-cm unstable bridge without the “step” and the 99-cm unstable bridge with the “step”.

For “crossings,” a significant trend was presented for “step” (*p* < 0.07; *F*_1,21_ = 3.733). Subjects cross the bridge without the gap more than the bridge with the gap (with need of a last step). Moreover, a significant trend was presented for a between-subjects effect (companion, *p* < 0.06; *F*_2,21_ = 3.111). Tukey HSD *post-hoc* test shows that control WT peer companion rats cross more than WT with DAT^trunk^ companion rats do (Figure 4). More than half of the latter rats did not cross at all, yielding overall to an average below one.

For “pawslips,” a significant trend was presented for “step” (*p* < 0.06; *F*_1,21_ = 4.079). Subjects slip more when they face the bridge with the step than the bridge without it. The ANOVA does not show any between-subjects significant effect.

For “turnabout,” a significant trend was presented (*p* < 0.09; *F*_1,21_ = 3.172). Subjects returned to the starting point more often when they faced the bridge with the step than the bridge without it. The ANOVA does not show any between-subjects significant effect.

For “latency,” the ANOVA shows a significant effect for “step” (*p* < 0.05; *F*_1,9_ = 9.406). Subjects cross the bridge without the gap significantly earlier than the bridge with the gap (with need of a last step). Moreover, the ANOVA shows a significant interaction for “step ^*^ companion” (*p* < 0.05; *F*_1,9_ = 5.720). Indeed, all groups crossed the bridge without the “step” with a lower latency.

## Discussion

In this study, we used an adapted protocol of a task previously developed in mice (Bortolato et al., 2009) to capture venturesomeness-related behaviors. Bridges of various kinds allowed rats to reach a food reward by making a choice: either take risks of falling in order to reach the food or waive the food and stay safe. Specifically, we explored the phenotypic differences of WT rats spending adolescence in different circumstances to understand if the companion of a diverse nature could influence the rats' risk-taking behavior.

We can affirm that all the subjects have shown rapid habituation to the apparatus, since they crossed both the 66 and the 99-cm bridges with shorter latency compared to the 33-cm one. Furthermore, due to such rapid habituation, subjects crossed the different bridges with lower latency on the 2nd day of exposure than on the first. Finally, they noticed the different bridges' length since they crossed the 99-cm bridge with a higher latency than the 66-cm one. Although they have become accustomed to the precarious situation, they hesitated to immediately cross the 99-cm bridge.

As for “stability” of the longer bridge, subjects crossed the stable bridge more times than the unstable one perhaps due to the oscillation of the latter. On this occasion, WT with adult companion rats slipped more often than WT peer companion and WT with DAT^trunk^ companion rats. A probable interpretation of this finding is that the latter was more scared and crossed more quickly, or did not cross at all; while the former took their time and crossed more calmly, suggesting some problems with motor coordination. In general, subjects crossed the unstable bridge with lower latency than the stable one, denoting again a quick habituation. However, they went back more often while crossing the unstable bridge probably because, at first, they did not expect it to swing. The fact that the unstable bridge was crossed with lower latency seems strange, but a possible explanation is that, as soon as they perceived it to swing and felt in danger, rats hurried up to complete the crossing, thus yielding overall to a lower latency.

Finally, all subjects crossed the bridge without the “gap” more often than the bridge with the gap and need of a last “step” probably because of the increased swing of the latter. Besides, subjects crossed the bridge without the “gap” in less time. When they were preparing to make the first step by placing the two forepaws on the bridge, they probably perceived the increased oscillation compared to the day just before, and this delayed their stepping and/or caused a turnabout. This gap-induced increased oscillation also explains the greater number of pawslips observed. Turnabouts are also particularly numerous on the bridge with the “step.”

WT with DAT^trunk^ companion rats immediately showed a restless environmental exploration. Compared to the other groups, the WT with DAT^trunk^ companion rats made fewer crossings on average, regardless of the length of the bridge. Moreover, even if they crossed the bridge, after eating some food pellets, they rarely went back through the bridge to the starting point, but they spent more time exploring the endpoint box. Avoiding the bridge, they displayed a risk-averse and more anxious behavior compared to both WT adult companion rats and control WT peer companion rats. A “restless exploration” is a typical feature of KO rats (Adinolfi et al., 2019); therefore, we can say that the observing of truncated-DAT rats by growing WT rats influenced the WT rats' developing phenotype. Besides, control WT peer companion rats crossed more than other groups on average. Particularly when they faced the unstable bridge, WT peer companion rats crossed significantly more than WT with DAT^trunk^ companion rats did, confirming the link between normal adolescence and enhanced risk-taking behavior (Gore-Langton et al., 2020).

Given the results, we can state that different types of companions influenced the development of WT rats' risk-taking behavior. WT peer companion rats showed a risk-taking behavior proclivity, while the WT with DAT^trunk^ companion rats seemed to feel unsafe, showing a continuous environmental exploration. Furthermore, it is interesting to highlight that WT peer companion rats continually crossed back and forth from the start point to endpoint and *vice versa* over and over, although the food reward was only in the endpoint, while the WT with DAT^trunk^ companion rats often did not cross at all, and rarely crossed back again to return to the start point—they preferred to stay at the end point to eat and greatly explore.

In our opinion, these apparatus and procedure could be useful to investigate risk-taking behavior. Indeed, to cross the bridge, each subject has to take the risk of falling because of a sudden bending of the bridge and cope with the feeling in danger for this unavoidable situation. In this context, such procedure can be combined with other tests to create a statistically valid battery of tests to study animal behavior, perception, and cognitive functions. As for limitations, the apparatus can be cumbersome, and the procedure can be dangerous to the animals. The experimenters should be careful in the choice of appropriate floor covering to avoid harm for the subject in case of fall.

In conclusion, we used this paradigm to investigate the risky behavior of rats and the influence that diverse companions at adolescence could have on it. We believe that the use of our “Himalayan Bridge” could be extended to model other behavioral anomalies like those observed in some human psychiatric disorders and cognitive dysfunctions.

## Data Availability Statement

The raw data supporting the conclusions of this article will be made available by the authors, without undue reservation.

## Ethics Statement

All experimental procedures have been approved by the ISS animal welfare survey board, on behalf of the Italian Ministry of Health (formal license 937/2018-PR and 1008/2020-PR for project D9997.110, delivered to WA). Procedures were carried out in close agreement with the directive of the European Community Council (2010/63/EEC) and with the Italian law guidelines. All efforts have been made to minimize the suffering of animals and to use as few animals as possible, according to the 3Rs principle.

## Author Contributions

MB designed the study. FF, CB, and AP carried out the behavioral experiment and then analyzed all behavioral data. FF and CB wrote the first draft of the paper together. WA, GL, MO, and GC commented critically. All authors together contributed to its final version.

## Conflict of Interest

The authors declare that the research was conducted in the absence of any commercial or financial relationships that could be construed as a potential conflict of interest.
